# Ten Simple Rules for Building and Maintaining a Scientific Reputation

**DOI:** 10.1371/journal.pcbi.1002108

**Published:** 2011-06-30

**Authors:** Philip E. Bourne, Virginia Barbour

**Affiliations:** 1 Skaggs School of Pharmacy and Pharmaceutical Science, University of California San Diego, La Jolla, California, United States of America; 2 Public Library of Science, Cambridge, United Kingdom

*While we cannot articulate exactly what defines the less quantitative side of a scientific reputation, we might be able to seed a discussion. We invite you to crowd source a better description and path to achieving such a reputation by using the comments feature associated with this article. Consider yourself challenged to contribute.*

At a recent Public Library of Science (PLoS) journal editors' meeting, we were having a discussion about the work of the Committee on Publication Ethics (COPE; http://www.publicationethics.org/), a forum for editors to discuss research and publication misconduct. Part of the discussion centered on the impact such cases have on the scientific reputation of those involved. We began musing: What on earth is a scientific reputation anyway? Not coming up with a satisfactory answer, we turned to a source of endless brainpower—students and other editors. Having posed the question to a group of graduate students, PLoS, and other editors, we got almost as many different answers as people asked, albeit with some common themes. They all mentioned the explicit elements of a reputation that relate to measurables such as number of publications, H factor, overall number of citations etc., but they also alluded to a variety of different, qualitative, factors that somehow add up to the overall sense of reputation that one scientist has for another.

What these students and editors identified en masse is one important side of a scientific reputation that is defined by data; but they also identified a much more nebulous side, that, while ill-defined, is a vital element to nurture during one's career. A side defined to include such terms as fair play, integrity, honesty, and caring. It is building and maintaining this kind of less tangible reputation that forms the basis for these Ten Simple Rules. You might be wondering, how can you define rules for developing and maintaining something you cannot well describe in the first place? We do not have a good answer, but we would say a reputation plays on that human characteristic of not appreciating the value of something until you do not have it any more.

A scientific reputation is not immediate, it is acquired over a lifetime and is akin to compound interest—the more you have the more you can acquire. It is also very easy to lose, and once gone, nearly impossible to recover. Why is this so? The scientific grapevine is extensive and constantly in use. Happenings go viral on social networks now, but science has had a professional and social network for centuries; a network of people who meet each other fairly regularly and, like everyone else, like to gossip. So whether it is a relatively new medium or a centuries-old medium, good and bad happenings travel quickly to a broad audience. Given this pervasiveness, here are some rules, some intuitive, for how to build and maintain a scientific reputation.

## Rule 1: Think Before You Act

Science is full of occasions whereupon you get upset—a perceived poor review of a paper, a criticism of your work during a seminar, etc. It is so easy to immediately respond in a dismissive or impolite way, particularly in e-mail or some other impersonal online medium. Don't. Think it through, sleep on it, and get back to the offending party (but not a broader audience as it is so easy to do nowadays with, for example, an e-mail cc) the next day with a professional and thoughtful response, whatever the circumstances. In other words, always take the high road whatever the temptation. It will pay off over time, particularly in an era when every word you commit to a digital form is instantly conveyed, permanently archived somewhere, and can be retrieved at any time.

## Rule 2: Do Not Ignore Criticism

Whether in your eyes, criticism is deserved or not, do not ignore it, but respond with the knowledge of Rule 1. Failure to respond to criticism is perceived either as an acknowledgement of that criticism or as a lack of respect for the critic. Neither is good.

## Rule 3: Do Not Ignore People

It is all too easy to respond to people in a way that is proportional to their perceived value to you. Students in particular can be subject to poor treatment. One day a number of those students will likely have some influence over your career. Think about that when responding (or not responding). As hard as it is, try to personally respond to mail and telephone calls from students and others, whether it is a question about your work or a request for a job. Even if for no other reason, you give that person a sense of worth just by responding. Ignoring people can take other serious forms, for example in leaving deserving people off as paper authors. Whether perceived or real, this can appear that you are trying to raise your contribution to the paper at the expense of others—definitely not good for your reputation.

## Rule 4: Diligently Check Everything You Publish and Take Publishing Seriously

Science does not progress in certainties—that is one of its joys but also what makes it such a hard profession. Though you cannot guarantee that everything you publish will, in 50 years' time, be shown to be correct, you can ensure that you did the work to the accepted standards of the time and that, whether you were the most junior or senior author, you diligently checked it (and checked it again…) before you submitted it for publication. As a first author you may well be the only one who appreciates the accuracy of the work being undertaken, but all authors have a responsibility for the paper. So, however small or big your contribution, always be upfront with your co-authors as to the quality and accuracy of the data you have generated. When you come to be a senior author, it is so easy to take a draft manuscript at face value and madly publish it and move on. Both actions can come back to haunt you and lead to a perception of sloppy work, or worse, deception. As first author, this mainly lets down your other authors and has a subtle impact on your growing reputation. As the senior author of an error-prone study, it can have a more direct and long-lasting impact on your reputation. In short, take publication seriously. Never accept or give undeserved authorship and in addition never leave anyone out who should be an author, however lowly. Authorship is not a gift—it must be earned and being a guest or gift author trivializes the importance of authorship. Never agree to be an author on a ghostwritten paper. At best these papers have undeclared conflicts of interest; at worst potential malpractice.

## Rule 5: Always Declare Conflicts of Interest

Everyone has conflicts of interest, whether they are financial, professional, or personal. It is impossible for anyone to judge for himself or herself how their own conflict will be perceived. Problems occur when conflicts are hidden or mismanaged. Thus, when embarking on a new scientific endeavor, ranging from such tasks as being a grant reviewer, or a member of a scientific advisory board, or a reviewer of a paper, carefully evaluate what others will perceive you will gain from the process. Imagine how your actions would be perceived if read on the front page of a daily newspaper. For example, we often agree to review a paper because we imagine we will learn from the experience. That is fine. Where it crosses the line is when it could be perceived by someone that you are competing with the person whose work you are reviewing and have more to gain than just general knowledge from reviewing the work. There is a gray area here of course, so better to turn down a review if not sure. Failure to properly handle conflicts will eventually impact your reputation.

## Rule 6: Do Your Share for the Community

There is often unspoken criticism of scientists who appear to take more than they give back. For example, those who rarely review papers, but are always the first to ask when the review of their paper will be complete; scientists who are avid users of public data, but are very slow to put their own data into the public domain; scientists who attend meetings, but refuse to get involved in organizing them; and so on. Eventually people notice and your reputation is negatively impacted.

## Rule 7: Do Not Commit to Tasks You Cannot Complete

It tends to be the same scientists over and over who fail to deliver in a timely way. Over an extended period, this becomes widely known and can be perceived negatively. It is human nature for high achievers to take on too much, but for the sake of your reputation learn how to say no.

## Rule 8: Do Not Write Poor Reviews of Grants and Papers

Who is a good reviewer or editor is more than just perception. Be polite, timely, constructive, and considerate and, ideally, sign your review. But also be honest—the most valued reviewers are those who are not afraid to provide honest feedback, even to the most established authors. Editors of journals rapidly develop a sense of who does a good job and who does not. Likewise for program officers and grant reviews. Such perceptions will impact your reputation in subtle ways. The short term gain may be fewer papers or grants sent to you to review, but in the longer term, being a trusted reviewer will reflect your perceived knowledge of the field. Although the impact of a review is small relative to writing a good paper in the field yourself, it all adds up towards your overall reputation.

## Rule 9: Do Not Write References for People Who Do Not Deserve It

It is difficult to turn down writing a reference for someone who asks for one, even if you are not inclined to be their advocate; yet, this is what you should do. The alternative is to write a reference that (a) does not put them in a good light, or (b) over exalts their virtues. The former will lead to resentment; the latter can impact your reputation, as once this person is hired and comes up short, the hirer may question aspects of your own abilities or motives.

## Rule 10: Never Plagiarize or Doctor Your Data

This goes without saying, yet it needs to be said because it happens, and it is happening more frequently. The electronic age has given us tools for handling data, images, and words that were unimaginable even 20 years ago, and students and postdocs are especially adept in using these tools. However, the fundamental principle of the integrity of data, images, and text remains the same as it was 100 years ago. If you fiddle with any of these elements beyond what is explicitly stated as acceptable (many journals have guidelines for images, for example), you will be guilty of data manipulation, image manipulation, or plagiarism, respectively. And what is more, you will likely be found out. The tools for finding all these unacceptable practices are now sophisticated and are being applied widely. Sometimes the changes were done in good faith, for example, the idea of changing the contrast on a digital image to highlight your point, but one always needs to think how such a change will be perceived and in fact whether it might, even worse, give the average reader a false sense of the quality of that data. Unfortunately, even if done in good faith, if any of these practices are found out, or even raised as a suspicion, the impact on one's career can be catastrophic.

In summary, there are a number of dos and don'ts for establishing a good reputation—whatever that might be. Do not hesitate in giving us your thoughts on what it means to be a reputable scientist.

